# Sexual education for university students and the community in a european project: study protocol

**DOI:** 10.1186/s12912-023-01350-5

**Published:** 2023-06-07

**Authors:** I. Soto-Fernández, R. Fernández-Cézar, A. M. Aguiar Frias, H. Dias, C. Santiago, C. Gradellini, V. Aaberg, V. García-López, S. Gómez-Cantarino

**Affiliations:** 1 EdSex Project, Diputación de Toledo, Residencia Social Asistida San José, Universidad de Castilla-La Mancha, Campus de Toledo, Nursing, Toledo, 45071 Spain; 2 EdSex Project, Facultad de Educación de Toledo, Universidad de Castilla-La Mancha, Toledo, 45071 Spain; 3 EdSex Project, Escola Superior de Enfermagem, Departamento de Enfermagem. Comprehensive Health Research Centre (CHRC), Universidade de Évora, Évora, Portugal; 4 EdSex Project, Escola de Saude, Politécnico de Santarém, Quinta do Mergulhão Srª da Guia, Santarém, 2005-075 Portugal; 5 Centro de Investigação sobre Tecnologias e Serviços da Saúde (CINTESIS), NursID – Inovação e Desenvolvimento em Enfermagem, Universidade do Porto, Porto, Portugal; 6 Centro de Investigação da Qualidade de Vida (CIEQV), Politécnico de Santarém e Politécnico de Leiria, Leiria, Portugal; 7 EdSex Project, Campus Universitario San Lazzaro, Università di Modena e Reggio Emilia, 2-42122 Vía Amendola, Reggio Emilia, Italy; 8 School of Health Sciences, Seattle Pacific University, 98119 Seattle, WA USA; 9 EdSex Project, Departamento de fisioterapia y terapia ocupacional, Universidad de Castilla-La Mancha, Campus de Toledo, 45071 Nursing, Toledo, Spain; 10 Health Sciences Research Unit: Nursing (UICISA: E), Coimbra Nursing School (ESEnfC), 3004-011 Coimbra, Portugal

**Keywords:** Health Education, Nursing, Professors, Sexuality, Student, Higher education, Migrants, Women

## Abstract

**Background:**

The World Health Organisation (WHO) calls on stakeholders to give Higher Education a key educational importance for the future of Europe. Within the content of the training programmes at university, sexuality emerges as a relevant topic in the nursing degree, to promote integral health from a holistic perspective. However, research on the presence of sexuality at the curricular level in Higher Education suggests that it is incomplete and underdeveloped.

**Methods:**

This is a protocol for a long-term, multi-centre, exploratory, descriptive, and cross-sectional study with a quantitative and qualitative approach lasting two years. The research will be carried out in the educational community, including, on the one hand, students, and professors and health professionals of nursing programmes from five universities in different parts of the world (Portugal, Spain, Italy, and the United States), and on the other hand, women, young people, and immigrants from these communities. The study will have several target populations. Firstly, the target is nursing students, with whom the aim is to define their perspective on the sexuality content taught at the university, and their level of knowledge. Secondly university professors and health professionals, with whom we will check their perspective on sexuality in the classroom, as well as their level of knowledge in this field. And finally, we will work with the community (women, young people, and immigrants) to whom we will try to bring sexuality from a useful and enjoyable perspective. In order to measure these variables in the protocol, instruments such as questionnaires and semi-structured interviews will be used. During data collection, ethical principles will be guaranteed and informed consent will be requested from the participants.

**Discussion:**

The results of the research will have a high curricular impact on the educational community, and will last over time, since the tools generated in the project will be included as part of nursing training programmes. In addition, participation in the project will improve health education for health professionals and at the community level on sexuality in both urban and rural populations.

## Background

Sexuality is a vital part of human beings with which we go along from infancy to senescence and helps us develop as people [1, 2]. It has an important physiological aspect but is also a form of personal expression [3], and everyone is unique in defining their sexuality with connotations that differ from one person to another [4, 5].

When sexuality is addressed in childhood, the family acts as the first source of learning. The values and beliefs of the legal tutors in this matter are influenced by social, cultural, and religious circumstances in every part of the world which will be reflected on the child’s education [6]. Some studies describe the factors that affect sexual education in children and teenagers. On the one hand, family act as a balancing factor in avoiding early sexuality practices in adolescence by impeding some unnecessary risky practices at this stage [7, 8]. On the other hand, the school is the place where freedom in sexuality can be promoted through a safe approach that is adapted to the maturity development of the child at each moment. The school environment is an appropriate setting for promoting sexual health in a holistic manner from the beginning of the person’s life cycle in early childhood education, as well as later in secondary education during adolescence [9]. In fact, there are educational laws in some countries that support comprehensive sexuality education from an affective-sexual perspective [10]. Sexuality is therefore a fundamental topic for students at school [11, 12]. In Europe, according to some research conducted in the adolescence, sexual education is based on just the recommendation of abstinence, safe sex based on contraception and on addressing personal growth in this area [13]. For this reason, it is important to address sexuality at an early age to avoid sexually transmitted infections (STIs) among other things and to deal with sexuality through assertiveness training [14, 15].

As early as 1975, the WHO in Geneva approached the concept of sexuality from the perspective of the health professional [16]. Sexuality should not be seen as a specific problem but from the point of view of promoting the health of the individual, and the health professional should embrace this dimension to provide holistic quality care [17]. In Australia, there is a speciality of sexual health for nurses, and it is evolving. It became evident that there was a need for a sexual health nursing model that addresses the two main themes of professional responsibility and patient care. The professional role incorporates a philosophy of sharing nursing experiences, collaboration, employment in multiple settings and development of the role in advanced practice, appropriate academic and clinical preparation, and a commitment to research. The patient care role includes the provision of individual and holistic patient care, the ability to access specific at-risk groups, clinical effectiveness, patient education and community development functions [18].

From an international perspective which recognises the great importance of skills and competences acquired through Higher Education (HE), the role of universities is becoming increasingly important. If we focus on sexual competence, it stands out that, in the teaching and learning process in HE in nursing students in part of the countries of the southern area of the European Union (EU) (Spain, Italy, Portugal), there is a standardised curricular dimension guided by a behaviourism based on a biological view of sexuality. Even the theoretical and clinical teaching of sexuality is approached from a reproductive health perspective with scarce and outdated content that does not attract the attention of students [19–21]. Besides, the lack of time and other content priorities place sexuality out of this set, rather on a second tier in nursing classrooms [22, 23]. Furthermore, considering that factors influencing sexuality are also socio-cultural, increased knowledge of sexuality could lead to interventions to improve nursing cultural competence [24]. Therefore, there is a clear need for sexual competence training that takes a more holistic view [25] and brings us closer to providing congruent care [26, 27] and adapted to diverse groups such as lesbian, gay, transgender, and bisexual (LGBT). It is important to increase sexuality self-awareness and knowledge of sexuality among nursing students and to reflect this in educational programmes [28]. An integrative review shows that nursing students’ attitudes towards LGBT in the 21st century are moving towards positive inclusion whereas in studies prior to 2000 the preponderance was negative towards this group [29]. In the same vein, the first National Summit of Nurses on LGBT Health in the USA agreed to increase sexual and gender minority (SGM) content in nursing curricula, practice guidelines, professional development, and research to improve the health of LGBT nurses, calling on the nursing profession [30]. Other research indicates that homophobia among health professionals, including midwives, has negative consequences for the health care of lesbian, gay and bisexual women [31]. To address that, for instance, in Spain an instrument has been developed to detect transphobic positions in the social and health care field [32]. In fact, social science points out that homophobia is culturally specific and constructed, so midwives are in a unique position to provide adequate professional services to lesbian women [33]. But the reality is that actually, there is a lack of training on sexuality of transgender people in the health care workforce, resulting in disparities of treatment in the health care setting [34, 35], in difficult adaptation of the health care provider with transgender patients, in limited care and increased risk for patients [36], with the result that health policy is not a moderating factor in the discrimination of gender minorities in this area [37].

With all the information reported above, it is highlighted that sexuality in HE exhibits a mismatch between the curricular contents that are currently taught and the knowledge and skills that the future nurse should ultimately have to provide quality care [23, 38, 39], even though the nursing student is highly qualified in other contents [40, 41]. This may be due to teachers’ discomfort in addressing sexuality content in the classroom, among other things [42].

In the health system, as health professionals, nurses should have skills to deal with sexuality for cardiac patients [43], oncology [44], stoma patients [45] and puerperal patients [20]. Nurses need training to improve care and ensure sexuality support to their patients, and to reduce sexual risks in them [46]. Some research reports that 80% of health professionals are unable to address sexuality in the workplace [17, 20, 21]. Paediatric nursing has long neglected the issue of sexuality, relying on myths that children are asexual. It is therefore important to establish a framework for sexuality education at this stage with practical advice for nurses and families [47].

Faced with a complex picture that leads to sexual health problems in the population, health policies should now promote sexual health promotion through the development of comprehensive approaches. Recently, there have been several attempts to develop such strategies at national, regional, and global levels. One of the milestones has been to define sex, sexuality, sexual health, and responsible sexual behaviour. The strategic thrust is for the health sector to take the lead in creating scenarios for discussion on sexuality, access to sexuality information and education, prevention strategies, access to health care, more research on human sexuality, and evaluation of programmes designed to promote sexual health and responsible sexual behaviour [48].

For all the above, this project is justified since it aims to carry out a cross-cultural and multidisciplinary training approach with the introduction of a comprehensive sexuality education model in Higher Education. In fact, this issue is so important that the Spanish National Agency for the development and management of the Erasmus + programme of the European Union in the field of Education and Training, such as the *Organismo Autónomo Servicio Español para la Internacionalización de la Educación* (SEPIE), promotes this type of initiatives and studies. In line with this, this project has been built in detecting a current need in students and teachers, and health professionals but also in the general population addressed through a practical training intervention to promote health following the lines of research, training and teaching innovation has been funded in the European Call for 2020–2021 with the code 2021-1ES01-KA220-HED-000023306. The need for this programme has been identified by both students and teachers, but also in healthcare practice, specifically in healthcare professionals. Not forgetting the general population, as this need is addressed through a practical intervention to promote health, reaching rural and urban communities, i.e. the general public. It also contributes to strengthening this in other social fields (youth, women, and immigrant associations), providing new visions in the field of sexual competence beyond our borders, and to the modernisation of sexual education in the socio-health field. The transnational approach becomes a basic requirement, since it promotes intercultural dialogue and enables awareness of a new Europe enriched by different cultures. In order to frame the approach in accordance with the needs of the project, the objectives are subdivided taking into account to the actors involved: (1) for students: increase knowledge; enhance critical thinking; acquire intercultural awareness/practical skills and constant practice; open up to sexual, social, gender and cultural diversity, acquire knowledge of existing and emerging norms regarding sexuality, and enhance the implementation of the knowledge, by fostering a sense of belonging to the EU; (2) considering teachers and health professionals: 2.1 for university professors: increasing sexual skills/competences; using innovative pedagogical approaches focused on learners, migrants, women and young people, in different modalities (face-to-face/distance) at international level; possibility of comparison/cooperation with other European universities; 2.2 for health professionals: to detect existing needs in the approach to sexuality in healthcare practice, to promote the development of guidelines that cover biopsychosocial care and to create strategies that allow for the analysis, diagnosis and approach to sexuality in patients in any healthcare setting.3) for the universities involved in the project: facilitating theoretical/practical sexual competence; possibility of mobility and cooperation between EU partners; developing innovative educational approaches (gamification); producing face-to-face/distance learning formats; strengthening EU inter-university networks. 4) for social and cultural groups: to promote intercultural outreach and respect at sexual level; to enhance critical thinking 5) for Bachelor of Nursing universities outside the project: to facilitate the free use of distance learning formats on sexuality; to promote the development of training on sexuality within the European nursing profile. 6) for Higher Education Degrees outside the project: to facilitate free access to distance learning formats on sexuality. This research will boost HE on sexual education internationally in the nursing programmes of the study member universities: the University of Castilla-La Mancha (Toledo, Spain), the University of Évora (Portugal), the Polytechnic Institute of Santarem (Portugal) and the University of Modena, Reggio Emilia (Italy) and Seattle (USA); 7) address a sex education counselling programme through training workshops that generate respect and a complete experience in the community, while meeting the identified needs of the rural and urban population; 8) establishing group mentoring by reaching out to the youth population from students in higher education to students in non-compulsory secondary education (pre-university), and from them to students in compulsory secondary education. Promoting respect for personal values and sexual orientations.

## Methods

The research is exploratory and descriptive with a quantitative-qualitative approach that will be carried out over a period of two academic years. Therefore, it is considered to be a long-term study of multicentric nature, as it has the following partner universities: (a) University of Castilla-La Mancha, Toledo, School of Nursing and Physiotherapy, Spain; (b) University of Évora, School of Nursing S. João de Deus, Évora, Portugal; (c) Polytechnic Institute of Santarem, School of Health of Santarem, Portugal, (d) University of Modena and Reggio Emilia, Italy. The research team has as guest university the University of Seattle, USA. These universities participating in the project have joined together in a consortium due to their involvement in the inclusion of sexual competence in the university studies of the bachelor’s degree in nursing. Because of its multi-centre nature this project can generate a considerable number of results and data. The title of the project is: “Educating in Sexuality: Advancing European Health”, which responds to the acronym EdSeX; the project will last for the academic years 2022–2023, and 2023–2024, with the main objective of describing the teaching of human sexuality in nursing programmes and analysing the context in which these contents are contemplated (or not). In addition to training teachers and healthcare professionals to provide biopsychosocial education and care that encompasses sexual health for the general population in urban and rural areas.

The specific objectives are:

1) to describe students’ attitudes and beliefs towards patients’ sexuality,

2) to profile students’ attitudes and beliefs about sexuality and sexuality education,

3) to define the students’ perception of their quality of sexual life,

4) to analyse students’ perspective on the recognition of human sexuality in the curriculum,

5) to describe professors’ and health professionals’ knowledge of sexuality.

6) to define professors’ theoretical-practical attitudes towards sexuality education in the nursing degree.

7) to develop a sexual education counseling program through training workshops in rural and urban populations.

8) to establish a cascade group mentoring program from higher education to secondary education, promoting personal values and sexual orientations.

The stated objectives will be achieved through intellectual outcomes, which are the items that must be fulfilled to carry out the research project. Four intellectual results have been set out, each assigned to one of the participating universities who will act as responsible institution by leading it, although all the universities will work and replicate in the same way in their campuses and communities. The project will be nurtured and will grow from the participant contributions that will raise in the periodic transnational meetings (online/face-to-face) held with all the partners and guest university, creating among them synergies around cultural and sexual competence. In addition, doctoral research studies related to gender and sexuality will be promoted.

**Fig. 1:**
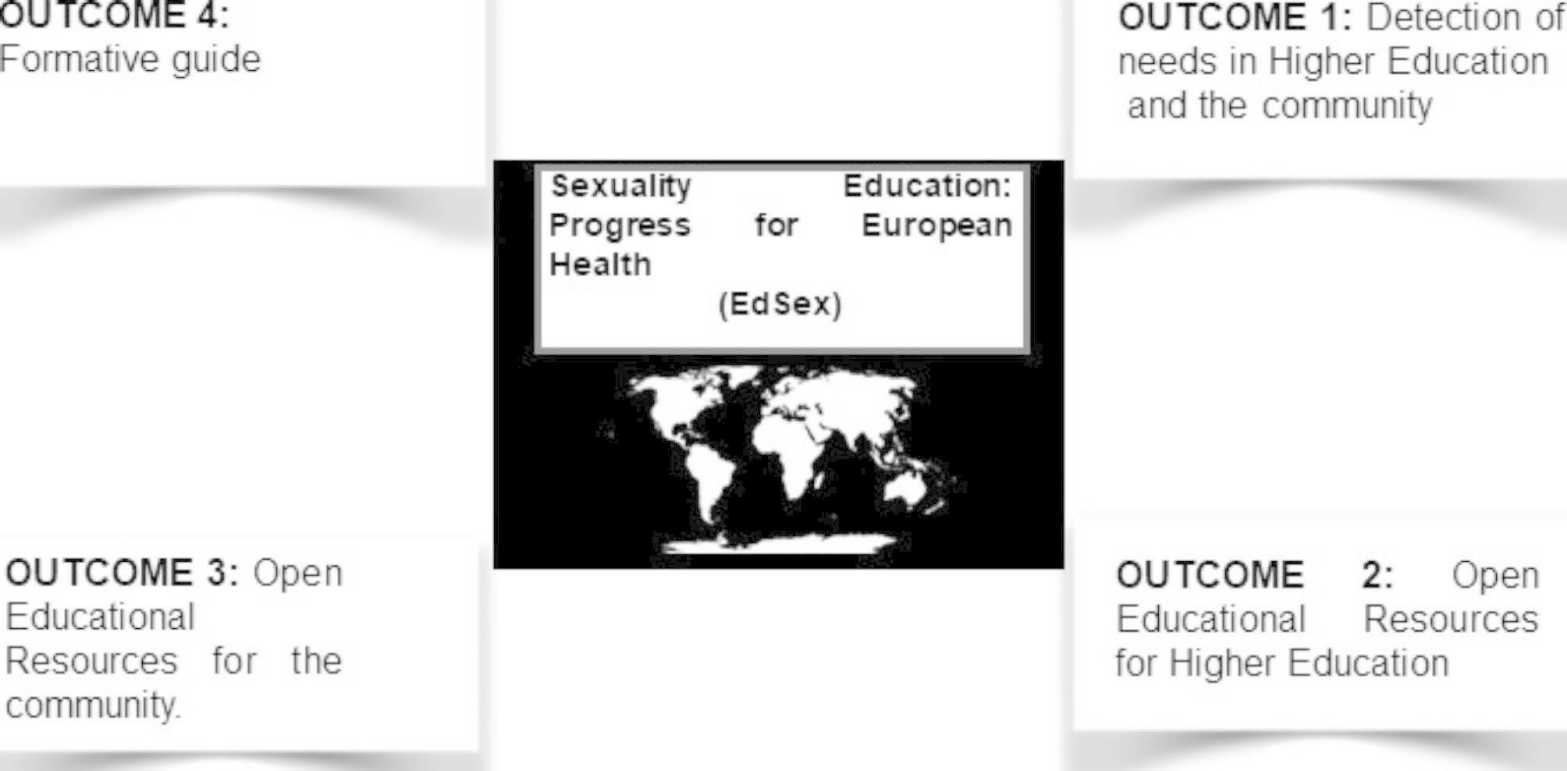
Project planning. Intellectual outcomes

The multicentre research protocol follow Helsinki Declaration has been submitted to the Social Research Ethics Committee (CEIS) of the University of Castilla-La Mancha (UCLM), in Toledo, Spain, and receives full approval (CEIS-661 803-V4Z4). The project will be addressed through the following outcomes, shown in Fig. 1:

**Outcome 1.** Research diagnostic: it is necessary to visualise the real needs and to define the actions and measures to be taken. The Unimore University (Italy) will elaborate a research report, which will include the state of the art on sexual competence, and that will consist of studies with complementary research designs. Thus, it will include a qualitative study on students and a quantitative study on university teachers and health professionals. These are detailed below:

O1.A) **Qualitative study** aimed at university professors and health professionals. The participants will **compose a convenience sample**, with each partner university committing itself to reach a number between 5 and 14 professors and health professionals. The sample is not exactly pre-determined, but it is expected to reach about 60 participants. Data saturation will show whether the sample size needs to be increased [40]. Potential participants will be both theoretical and practical teachers at the partner universities from hospitalisation, primary health care and social-health care settings, not forgetting school nursing. The professors will be selected on the basis of their profile of teaching students on the subject, taking into account the following inclusion criteria: (a) to be linked to the university with theoretical-practical training; (b) to be linked to training in the 2nd, 3rd, 4th academic year; (c) to have been a nursing teacher for at least 2 years; (d) to be a native speaker of the language of each university/centre/school where the data collection will be carried out. And the exclusion criteria will be: (a) novel professor in his/her first year of teaching; (b) teaching less than 3 h per week. Healthcare professionals will be selected based on: (1) the unit where they provide their clinical practice; (2) a minimum of 5 years of professional experience; and (3) the requirement that the healthcare unit where they provide services is affiliated with a university center. Exclusion criteria will include: (1) healthcare professionals not linked to direct patient care; (2) healthcare professionals in a period of clinical practice. Finally, the total sample will range between 50 and 60 participants (UCLM 14, Seattle 5, Santarem 14, Évora 12, Italy 12).

The instrument will be a semi-structured interview distributed in 3 blocks, adapted from Albano Estrela [49], where the first block includes socio-demographic data (age, social status, family status…), the second is composed by professional issues (training, experience…) and the third block deals with knowledge about sexuality (human sexuality, expression of sexuality content in the classroom, the professional’s perception of the students’ comments…).

**The procedure** with each of the informants will consist of the arrangement of an appointment (specific date and time, specific place), in an atmosphere that is pleasant and close to the participant in both the health and university domains, defined with them to favour communication. The estimated time for conducting the interview will range between 20 and 35 min. The data will be collected by recording the interview with the prior consent of the participants, guaranteeing their confidentiality and anonymity. At the end of the session, the audiotape will be transcribed, considering all the speech of the participants. The data will be entered into the NVivo programme by the teacher responsible for the project in each centre. The analysis of the data will be carried out by coding maps, dendrograms and Jaccard analysis.

O1.B) Pre-post quasi-experimental design study with a quantitative approach aimed at detecting the needs of higher education students: **the participants** will be people enrolled in the Nursing Degree in 2nd, 3rd and 4th academic year, for whom the following inclusion criteria will be taken into account: a) being enrolled in-person in the academic year; b)

having taken the subject of Theoretical Foundations of Nursing cultural diversity and maternal sickness; c) age between 18 and 34 years; d) being a native speaker of the language of each university institution in which the data collection will be carried out, e) to be enrolled in the sexual education workshops offered at each university. As exclusion criteria: (a) students over 35 years; (b) students enrolled in the distance learning modality, and (c) students enrolled in the Erasmus programme.

In terms of the number of students participating in the project, we expect to involve more than 200 students, so with a confidence level of 95% and a margin of error of 5%, the sample size should be N = 132, but it would be increased by 5% to counteract eventual incorrect and incomplete forms. Since there are 4 partner universities (Ni = N1, N2, N3, N4) and one guest university (N5), the commitment is different, so we assume a sample size of 25–30 per partner university and 20–25 for the guest university.Applying the criteria of Almeida et al. [50], the total sample, n, will be of about 132 students. Sampling process will be stratified by considering each university, with a proportional distribution for the stratus sample size by using the formula:

n_i_ = n x (N_i_/N).

The same proportionality rule will be applied for each academic year. The sample thus constituted has students enrolled from different areas of each country, so that their socio-cultural characteristics are representative of the participant universities.

### Procedure

The administration procedure will consist of providing a questionnaire to students who agree to participate in the study, either on paper or online. The confidentiality of the records will be guaranteed, and they will be collected anonymously.

### Instruments

In this study, the instrument will be a questionnaire composed by socio-demographic data and different scales. On the one hand, the Sexuality Attitudes and Beliefs Survey (SABS) [51] and in its Spanish version [52], Portuguese [53] or Italian [54] versions, will be used as instrument, previously validated by the partner universities. This SABS will reinforce the possible results obtained in this protocol. On the other hand, the Family Apgar Scale [55], the Attitudes and Beliefs about Sexuality and Sex Education Scale (QACSES) [56], the Male (SQoL-M) [57] and Female (SQoL-F) Quality of Sexual Life [58]. For this purpose, permissions have been achieved from the authors to use each of the above scales.

The SABS questionnaire offers as response options a Likert scale with six scores (from strongly disagree to strongly agree). Regarding the SABS scale, the nominal response range for the instrument is between 12 and 72 points: higher scores indicate a lower ability to handle the sexual dimension in nursing practice. The scale is unidimensional, and the original form has a Cronbach’s alpha coefficient of 0.750, and a test-retest of 0.820 [51]. The Portuguese version has been validated by removing one item (N3) that improves internal consistency for social sciences, reaching a Cronbach’s alpha coefficient of 0.720, and in the test-retest of 0.800 [53], while the Spanish study reported a Cronbach’s alpha coefficient of 0.651 and is finally maintaining the 12 items [52]. The validated instrument in the Italian version shows adequate internal consistency (alpha = 0.76) [55]. Overall, the validation process confirms the validity and reliability of the instrument in the different languages and contexts for which it has been validated, which guaranties their use in this project.

The Family Apgar Scale [55] is a tool for the evaluation of the level of functioning within the family core [32]. It is composed of five items assessing five domains (1) Intrafamilial Adaptability, (2) Association, (3) Growth, (4) Affect and (5) Resolution, together corresponding to the acronym of the five letters of “APGAR”. Each question presents three options: (2) almost always, (1) sometimes and (0) almost never. Scores range from 0 to 10 in the categories of: (1) Dysfunctional (score 0 to 3 points), (2) Moderately Dysfunctional (score 4 to 7 points), (3) Highly Functional (score 8 to 10 points) [25, 32]. Cronbach’s alpha was 0.80 in a Brazilian study. The discrimination coefficient ranged from 0.52 to 0.68 and the criterion validity showed a correlation coefficient of 0.76 [32].

Another instrument used in the research is the Attitudes and Beliefs about Sexuality and Sex Education Scale (QACSES) [56], a questionnaire composed of 17 items, scored on a 5-point Likert-type response scale (1 “strongly disagree” to 5 “strongly agree”). The scale has three dimensions: (1) Beliefs related to gender and contraception (items 4, 5, 6, 7, 11 and 13); (2) Beliefs associated with dating, gender, and sexual violence (items 1, 2, 3, 8, 9, 12 and 17); and (3) Beliefs associated with a relationship (items 14–16). Items 10, 16, 20, 21, 22 and 23 should be reversed for rating. Higher values represent more limiting beliefs and negative attitudes about sexuality and sex education. The internal consistency of the instrument’s subscales revealed Cronbach’s alpha indices between 0.72 and 0.75.

Finally, the Female Sexual Quality of Life Questionnaire (SQoL-F) [58] includes 18 items, each requiring a 6-point Likert-type response (1 strongly agree and 6 strongly disagree). A high score implies a better quality of sexual life. The score is obtained from the sum of the items after reversing those that are positively worded (items 1, 5, 9, 13, 16 and 18). In the original study, Cronbach’s Alpha coefficient was 0.950 [34].

The Male Sexual Quality of Life Questionnaire (SQoL-M) [57] is a modified version of the SQoL-F, which assesses quality of life in men and contains 11 items. The items have a 6-point Likert-type response, (1 strongly agree to 6 strongly disagree). A high score indicates a higher quality of life. The results are easily compared with other measures of quality of life using the following standardisation formula:

Scale score = ((Sum of real items - possible MIN)/(MAX - MIN)) × 100.

The SQOL-M is a unidimensional instrument with excellent internal consistency demonstrated by a Cronbach’s alpha coefficient of 0.82. In convergent validity, the SQOL-M correlated with the Premature Ejaculation Index, satisfaction (0.59) and distress (0.50), also exhibiting a good discriminant validity [37].

**Outcome 2.** Creation of Open Educational Resource, OER-EdSex, for Higher Education.

This result is divided into two training blocks, which in turn are subdivided into two modules, each with an epicentre topic to be dealt with on sexuality that will be given in a staggered manner over time in each of the universities involved in the Project. The target population to which this training is addressed is the Nursing Degree students at the universities involved, and the expected attendants in each of the universities will range from 10 to 30 students, except for the guest university (Seattle) which will count on the participation of at least 6 students. This training programme is designed as workshop for small groups to gather the attention of attendants rather than in large ones. Therefore, the access will be open only to a small number of participants. In this vein, the recruitment process will be carried out through posters as well as in the classrooms through the participant professors.

**Fig. 2:**
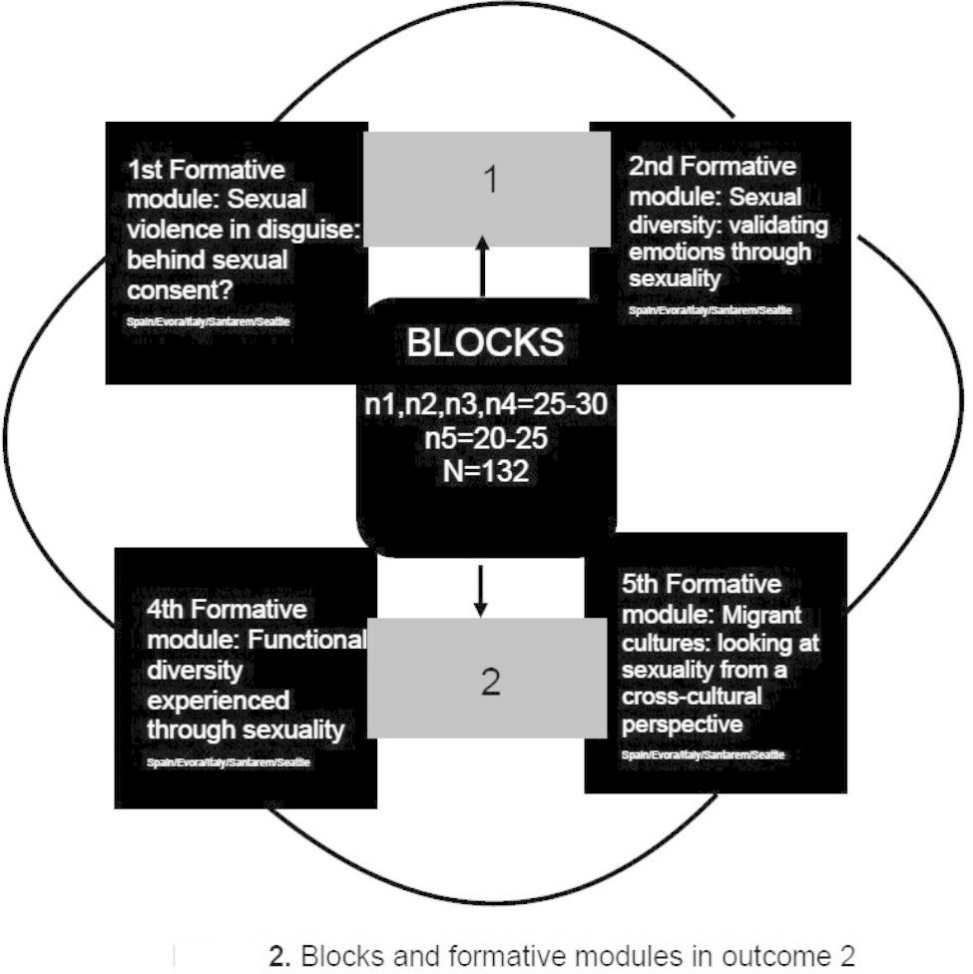
Blocks and formative modules in outcome 2

Each module will last 60–80 min and will be scheduled on a specific day of the week in consecutive months, starting in January 2023 and ending in May 2023. The foundation of healthy and holistic sexual education will be worked on, as it is a crucial aspect in the educational process, and the group composition will be cared to warranty inclusion and diversity. In this case, the group of students from each university is the main protagonist and agent of health education, and their participation in the development of the modules is another key element of this educational training (see Fig. 2).

Block 1 consists of two modules: (a) the 1st Training Module which deals with the main topic of Covert sexual violence: Behind sexual consent? and will be taught in January 2023 to all partner universities by expert teaching staff from the guest university in Seattle. They will be connected via Teams and the teaching session will be both synchronous at the convened time, and asynchronous. To keep the sessions, it will be recorded and posted on the Web-EdSeX platform to reach the widest possible dissemination. (b) The 2nd Training Module addresses the topic of Sexual Diversity: validating emotions from sexuality and will be taught in February 2023 by each partner university. Expert faculty/staff will be available to carry out the planned activities.

Block 2 is subdivided into two modules: (a) the 4th Training Module which deals with Functional Diversity experienced through sexuality and will be taught in March 2023 by Reggio Emilia staff to all partners. They will be connected via Teams and the teaching session will be both synchronous at the convened time, and asynchronous. The recorded sessions will be posted on the Web-EdSeX platform in order to reach the widest possible dissemination; (b) the 5th Training Module: Migrant Cultures: looking at sexuality from a transcultural perspective will be held in May 2023 by each partner university. Expert faculty/staff will be available to carry out the planned activities.

Once developed, the Open Educational Resource (OER-HE) will be hosted within the Web-EdSex website, where the dissemination of content in the field of sexuality in HE will be guaranteed for free. The UCLM will assess the contents to be included and will manage the development of this product, following consensus with all the participating universities and the invited one. Intellectual outputs 1, 2 and 3 are two fundamental axes of the project that are intertwined to have a greater impact on sexuality education at a multicentric level.

**Outcome 3.** Creation of Open Educational Resource, OER-EdSex, for the community.

**Fig. 3:**
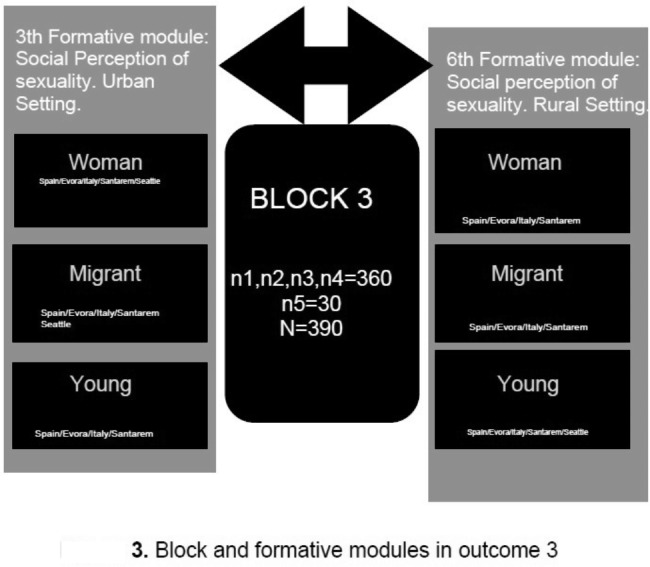
Block and formative modules in outcome 3

Like in outcome 2, this result is found in Training Block 3, which comprises 2 Training Modules (Module 3 and Module 6).which will be developed around a central theme on sexuality and will be given sequentially over time in each of the communities (urban and rural) where the partner universities are placed. In this sense, different youth, immigrant, and women’s associations will be contacted as participants, and educated. The number of participants will be between 10 and 15. Recruitment will be carried out through the Principals of Secondary Education schools in both urban and rural areas and by contacting the Primary Health Care management of the specific area, as well as non-governmental organisations. The aim of this training is to encourage individuals to take responsibility for themselves by actively participating in the process of generating knowledge and taking practical action on the conditions that affect their sexual health (Fig. 3).

In this 3rd Block, module 3 is composed of the Social Perception of sexuality and will be delivered in the urban environment and disseminated in the community during January/February/March 2024 to women, immigrants, and youth associations. The invited university will develop this module addressed to women in March 2024 with the aim of achieving the widest possible dissemination reaching the largest number of the target population.

The 6th module, deals with the Social Perception of sexuality in the rural environment, being disseminated in the community during April/May/June 2024. The invited university will develop this module aimed at young people in June 2024 with the objective of reaching the target population. To this end, contacts will be made with secondary and/or primary schools.

The Open Educational Resource (OER-Community) will be hosted in the Web-EdSex site in such a way that can be used and disseminate content in the field of sexuality to the community. The University of Évora will manage the and assess the development of this resource, after consensus with all the participating universities and the invited university. It will be developed by teachers and experts from all the partner universities and in this case, it will include students who wish to collaborate in community training through group mentoring, both at urban and rural level, as well as the community to which it is specifically addressed.

**Outcome 4.** Creation of formative guide.

The training guide will be generated to bring together the previous outcomes as a final product of the project (outcome 1, 2 and 3). It will be a tool for the acquisition of skills, habits, attitudes, and abilities oriented towards learning, resulting in the development of healthy sexual health in today’s society. The content will be adapted to all audiences and the language used will be accessible, inclusive and based on respect and freedom.

It will be included in the Web-EdSeX site and will be launched after the development of at least part of outcome 1 (needs detection) and two training modules corresponding to outcome 2. The University of Santarém will manage the contents to be included, following consensus with the participating universities and the invited university.

The Training Guide will be accompanied by formative research, as it will include strategies, contexts and interactions as a way of dynamizing the teaching-learning process, for example, with gamification where the rules of the game itself are part of the training.

This guide aims to generate a crucial product on this protocol and will be promoted in online and paper format to facilitate and encourage its use in the general population. This document will serve as a guide for future research.

## Discussion

This project aims to create an open resource to be used both in higher education and in the community to promote healthy and up-to-date sexuality education. This general objective will be reached through the achievement of the four outcomes presented.

The outcomes of this project will enhance the development of sexuality knowledge in multiple academic settings, both in higher education and pre-university levels, as well as in the community. On the one hand, this project is focused on nursing curricula, which will contribute to a high level of knowledge and skills for the provision of evidence-based sexual health care to future nurses. Therefore, nursing education can strengthen and improve sexuality content as part of university studies [21], acting as a transmitter of knowledge and promoting student-centred and culturally congruent learning [25, 26].

On the other hand and given that the professional training model [42] should be retrained with active learning about sexuality and include the development of the capacity to address sexual content with patients [59, 60], this project will contribute to the retraining of professionals already in service, creating appropriate synergies for both parties. There are models of care described by theorists such as Virginia Henderson or Hildegard Peplau, which provide tools for assessing sexuality and reproductive development of individuals, so that professionals have useful instruments in their daily work. Marjory Gordon’s Functional Health Patterns are also very useful in health care practice. These patterns configure behaviours common to all people, which contribute to their health, quality of life and the achievement of their human potential, which will occur in a sequential manner over time. Pattern 9, sexuality-reproductive, describes patterns of satisfaction or dissatisfaction with sexuality, and understands sexuality as the behavioural expression of sexual identity. It includes, but is not limited to, sexual relations with a partner. Cultural norms regulate its expression. Problems can arise when discrepancies occur between the expression of sexuality that the person has achieved and the one which he or she desires. There are also the Nursing Diagnoses of the American Society for Nursing Science. These allow nurses to analyse the sexual dimension of the patient with greater depth and relevance [61, 62]. Although nursing attaches great importance to sexuality, human sexuality is not yet perceived as an area of nursing [54].

To carry out this international multidisciplinary and multicentre study is a key point for sexual education, as it will provide a scientific analysis that will give us real clues about the state of sexuality education in the nursing profession and will help the integration of sexuality education in nursing education in several European countries directly involved [54]. It will contribute to the development of appropriate sexuality education, a crucial part of preparing nursing graduates to provide holistic care [17].

Its usefulness in improving the sexuality education of the present and future nursing profession is reinforced by the fact that nurses in a health care system are health agents capable of promoting the sexual and reproductive health of the population, as well as of preventing diseases associated with sexuality from all perspectives of the human being, including psychological, social, and others. Thus, nursing principles in the exercise of care are a prerequisite for the improvement of human sexuality, because through early health care and an integral intervention centred on health education involving the participation of the population, the integral sexual education of society could be promoted [63, 64].

For all these reasons, we must be committed to a society with adequate sexual health based on the training of nurses as the epicentre of such a task, as this research project on sexuality outlines. Without neglecting the fact that the education and positive promotion of healthy sexuality must involve the adoption of a holistic approach applied to the study of needs, planning, implementation, and evaluation of education programmes, designed so that future professionals and health professionals can efficiently reach different social groups.

## Data Availability

This paper is a study protocol so there are not direct data generated. Nonetheless, information on project development can be addressed through correspondence author (raquel.fcezar@uclm.es) and funding responsible person (sagrario.gomez@uclm.es ).

## Abbreviations

STI: Sexually Transmitted Infection
HE: Higher Education
EU: European Union
LGTB: Lesbian, Gay, Transgender, Bisexual and Transsexual
USA: United States of America
MSG: Sexual and Gender Minorities
EdSeX: Project Acronym
OER: Open Educational Resource
UCLM: University of Castilla−La Mancha
